# Olecranon Bursitis Caused by* Candida parapsilosis* in a Patient with Rheumatoid Arthritis

**DOI:** 10.1155/2016/2019250

**Published:** 2016-08-09

**Authors:** Carla F. Gamarra-Hilburn, Grissel Rios, Luis M. Vilá

**Affiliations:** Division of Rheumatology, Department of Medicine, University of Puerto Rico, Medical Sciences Campus, San Juan, PR 00936-5067, USA

## Abstract

Septic bursitis is usually caused by bacterial organisms. However, infectious bursitis caused by fungi is very rare. Herein, we present a 68-year-old woman with long-standing rheumatoid arthritis who developed pain, erythema, and swelling of the right olecranon bursa. Aspiration of the olecranon bursa showed a white blood cell count of 3.1 × 10^3^/*μ*L (41% neutrophils, 30% lymphocytes, and 29% monocytes). Fluid culture was positive for* Candida parapsilosis*. She was treated with caspofungin 50 mg intravenously daily for 13 days followed by fluconazole 200 mg orally daily for one week. She responded well to this treatment but had recurrent swelling of the bursa. Bursectomy was recommended but she declined this option. This case, together with other reports, suggests that the awareness of uncommon pathogens, their presentation, and predisposing risk factors are important to establish an early diagnosis and prevent long-term complications.

## 1. Introduction

Septic bursitis occurs in the olecranon bursa very commonly [1]; this is due to its superficial location and vulnerability to trauma [1]. Among predisposing factors for septic bursitis are immunosuppression, surgical intervention, chronic diseases, and any occupation that can produce trauma to the area [1, 2]. Bacteria are the most frequent culprit, with* Staphylococcus aureus* producing the majority of the cases [1, 2]. Fungal infection is rare and there are only few cases published in the medical literature. Septic bursitis due to* Candida* (*parapsilosis*,* albicans*,* tropicalis*, and* glabrata*) species,* Penicillium* species,* Anthopsis deltoidea*,* Aspergillus terreus*, and* Phialophora richardsiae* has been reported [1–13]. Herein, we report a case of an elderly woman with long-standing rheumatoid arthritis (RA) who presented with septic olecranon bursitis secondary to* Candida parapsilosis*. We also present a literature review of bursitis caused by* Candida* species.

## 2. Case Presentation

A 68-year-old female was referred to our service for evaluation of erythema and swelling of the right elbow. She was admitted to the hospital 12 days before because of an infected left foot ulcer that required debridement and intravenous antibiotic therapy. Six weeks prior to admission she was seen by a pain management specialist for swelling of the right elbow and she was diagnosed with olecranon bursitis. She was treated with bursa aspiration and corticosteroid injection. Cultures were reportedly negative. One to two weeks after the procedure, she had worsening of swelling, as well as pain and erythema of the olecranon bursa. She did not report fever, weight loss, fatigue, or weakness.

Her past medical history was remarkable for RA diagnosed at 30 years of age, arterial hypertension, peripheral vascular disease, and asthma. Her surgical history included amputations of the first and second toes of the left foot and two tendon repairs (left hand and left shoulder). She received multiple therapies for RA including auranofin, methotrexate, leflunomide, and etanercept. Her last treatment was golimumab, which she used for 4 years. She did not have further follow-up with her rheumatologist and stopped this treatment about 2 years before onset of olecranon bursitis.

**Figure 1:**
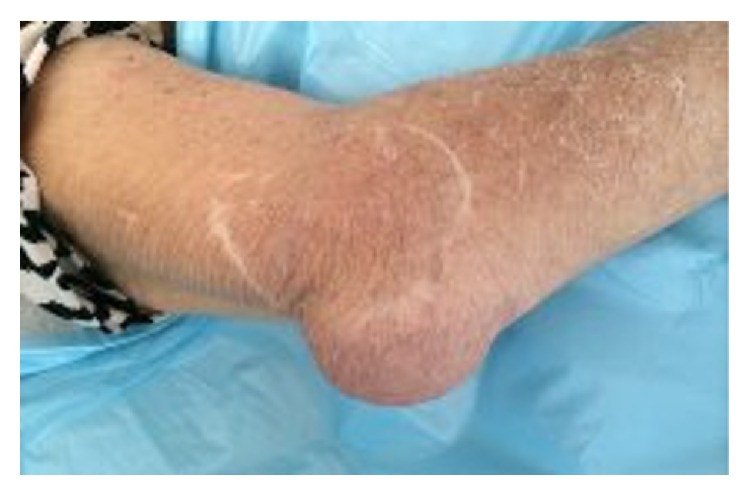
Erythema and swelling of the right olecranon bursa.

On initial evaluation, she had normal vital signs. Scleromalacia was noted bilaterally. Examination of the upper extremities showed ulnar deviation of the metacarpophalangeal joints and swan neck deformities bilaterally. The left wrist had marked limitation of flexion and extension. The right olecranon bursa was tender and swollen, and the overlying skin was erythematous (Figure 1). Range of motion of the right elbow was not limited. She had multiple subcutaneous nodules in the right leg and a stage IV ulcer on the medial aspect of the left foot. Generalized muscle atrophy was observed. The rest of the examination was unremarkable.

Laboratory tests showed a white blood cell count of 6.8 × 10^3^/*μ*L, lymphocytic count of 1.2 × 10^3^/*μ*L, hemoglobin of 9.6 g/dL, and platelet count of 344 × 10^3^/*μ*L. Serum creatinine was 0.5 mg/dL and blood urea nitrogen was 17 mg/dL. Wound cultures were negative. Chest X-ray showed mild prominence of the pulmonary markings in the right infrahilar region.

The olecranon bursa was aspirated and 9 mL of hemorrhagic fluid was obtained. No crystals were observed under polarized microscopy. Fluid analysis showed a white blood cell count of 3.1 × 10^3^/*μ*L (41% neutrophils, 30% lymphocytes, and 29% monocytes). Bacterial cultures were negative. Fungal culture was positive for* Candida parapsilosis*; this was sensitive to anidulafungin, micafungin, caspofungin, 5-flucytosine, voriconazole, itraconazole, and fluconazole. The VITEK 2 system (bioMérieux, Inc., Hazelwood, Missouri, USA) was used for fungus identification. The Sensititre YeastOne system (Trek Diagnostic Systems, Cleveland, Ohio, USA) was utilized to determine antifungal susceptibility.

She was treated with caspofungin 50 mg intravenously daily for 13 days. Four aspiration procedures were required (every 3–5 days) due to fluid reaccumulation. All samples were cultured but only the first two were positive for* Candida parapsilosis.* After intravenous therapy, fluconazole 200 mg orally daily was prescribed for one week. She responded well to this therapy but had recurrent swelling of the bursa. Bursectomy was recommended but she did not wish to proceed with the intervention or receive further treatment. After 3 months of follow-up, she continued to have fluid in the olecranon bursa and had some discomfort in the area.

## 3. Discussion

Symptoms and signs of septic bursitis, either bacterial or fungal, are similar. However, fungal bursitis seems to have a more indolent course which may cause a delay in diagnosis and treatment [2, 4–7]. Among all bursae, the olecranon and the prepatellar bursae are the most commonly involved; this is because they are superficial, and pathogens can migrate transcutaneously through minor trauma [2]. Although septic bursitis is almost always secondary to direct inoculation, hematogenous spread can also occur [8, 9].

Fungal septic bursitis is rare.* Candida* (*parapsilosis*,* albicans*,* tropicalis*, and* glabrata*),* Penicillium* species,* Anthopsis deltoidea*,* Aspergillus terreus*, and* Phialophora richardsiae* have been isolated [1–13]. Specifically, very few cases of olecranon bursitis secondary to* Candida parapsilosis* have been reported in the medical literature [3, 7, 10, 11]. Unlike* Candida albicans* and* Candida tropicalis*,* Candida parapsilosis* is not an obligate human pathogen [14]. The clinical spectrum of* Candida parapsilosis* infection is variable, ranging from vulvovaginitis to fungemia and endocarditis. It is also known to cause arthritis which can be secondary to fungemia, intra-articular injections, or catheterizations or related to prosthetic joints [8, 9, 11, 15].

To the best of our knowledge, 11 cases of septic bursitis due to* Candida* species, including ours, have been reported in the medical literature (Table 1) [1, 3, 4, 7–13]. Five cases, including our case, were secondary to* Candida parapsilosis* [3, 7, 10, 11]. Two cases were secondary to* Candida tropicalis* [8, 9], and the other three were due to* Candida albicans* [1], Candida* lusitaniae* [13], and* Candida glabrata* [12] (one each). The subclass identification of* Candida* species was not done in one patient with subacromial bursitis and the diagnosis was achieved histopathologically after bursectomy was performed [4]. Regarding the site involved, the olecranon bursa was affected in 7 of the 11 cases including our patient [3, 7, 8, 11–13]; the subacromial bursa was affected in 3 patients [1, 4, 10]; and the popliteal bursa was affected in 1 case [9]. Six patients were immunosuppressed and 1 patient was receiving prednisone at low dose (10 mg daily) [1, 7–9, 11–13]. Our patient had severe joint damage from RA, but she was not receiving immunosuppressive therapy at the time of septic bursitis.

In the 11 cases reported with* Candida* bursitis, the probable source of infection was thought to be fungemia in 3 cases [1, 8, 9], one case was thought to be secondary to superficial trauma [13], and in 3 cases the source of infection was undetermined [7, 10, 11]. Four cases were likely related to corticosteroid injection, including our case [3, 4, 12]. In our patient, although her musculoskeletal deformities and her duties as an administrative assistant predisposed to continuous trauma to the area, her symptoms worsened after the intrabursal injection. This event suggests that the pathogen could have been inoculated during the aspiration and corticosteroid injection. She could have also acquired the infection through transient fungemia from the wound in her foot. However, wound cultures were negative.

The course of septic bursitis due to fungi can be chronic and this delays diagnosis and treatment. Six of the 10 reported cases had a chronic course ranging from 4 to 18 months [3, 4, 8, 10, 13]; the remaining cases had an acute onset [1, 7, 9, 12]. In one case, the duration of the disease was not described but it presented 7 months after infliximab therapy was started [11]. Regarding the age at presentation, more than 70% of the patients were older than sixty years of age, this including our patient.

Fungal bursitis is treated with oral or parenteral antifungals as well as drainage of infected bursal fluid; this is frequently done by needle aspiration. Bursectomy is indicated when there is no response to this treatment, when the bursal fluid cannot be drained with a needle, or when there is infection of the surrounding soft tissue [2]. All of the patients that we describe in Table 1 received antifungal therapy at some point, but 7 required bursectomy to achieve cure [3, 4, 8–12]. Two patients were cured with antifungal therapy alone [1, 7]. One of the patients, who had olecranon bursitis due to* Candida lusitaniae*, was severely immunosuppressed, had recurrent bursitis, and ultimately died of* Pneumocystis jirovecii* infection [13]. Our patient had reaccumulation of fluid and bursectomy was indicated but she declined this option.

In summary, fungal infection due to* C. parapsilosis* is rare and follows an indolent course. Immunosuppression and advanced age appear to be predisposing factors for bursitis caused by* Candida* species. Septic bursitis caused by bacterial organisms usually responds well to antimicrobial therapy and fluid aspiration. In contrast, this therapeutic approach is not entirely effective for fungal bursitis caused by* Candida* species as recurrent bursitis is quite common. Definite cure is attained with bursectomy for which this procedure should be highly considered early in the management of these cases.

## Figures and Tables

**Table 1:** Demographic features, clinical manifestations, treatment, and outcome of septic bursitis caused by *Candida* species.

Case number	*Candida* strain/culture site	Author/year of publication	Age/sex	Infected bursa	Clinical presentation of bursitis	Comorbidities	Other risk factors	Probable source of infection	Treatment	Outcome
1	*C. albicans* Bursal fluid	Rosochmann and Bell/1987 [1]	73/M	Subacromial	Acute, 5 days	SLE	Corticosteroids	Fungemia	Amphotericin B	Complete resolution

2	*C. glabrata* Phlegmon-like material form olecranon bursae	Skedros et al./2013 [12]	63/F	Olecranon	Acute, 2 weeks after corticosteroid injection	COPD Arterial hypertension Hypothyroidism Recurrent oropharyngeal candidiasis	Prednisone 10 mg daily	Corticosteroid injection	Caspofungin Debridement, irrigation, and bursectomy	Complete resolution

3	*C. lusitaniae* Bursal fluid	Behar and Chertow/1998 [13]	59/F	Olecranon	Chronic, 6 months	SLE Diabetes Asthma	Methotrexate 15 mg weekly Prednisone 30 mg daily	Superficial trauma	Fluconazole 100 mg a day; 5-fluorocytosine	Recurrence after several months

4	*C. parapsilosis* Bursal fluid	Schlesinger and Hoffman/1995 [7]	62/F	Olecranon	Acute	Breast cancer COPD	Prednisone 40 mg daily	Undetermined	Amphotericin B IV over 9 days Ketoconazole (400 mg/d for 2 months)	Complete resolution

5	*C. parapsilosis* Bursal fluid	Jiménez-Palop et al./2002 [3]	32/M	Olecranon	Chronic, around 3 months	None	None	Corticosteroid injection	Fluconazole 400 mg for 7 days, followed by 200 mg a day Bursectomy	Complete resolution

6	*C. parapsilosis* Bursa tissue culture	Miyamoto et al./2012 [11]	60/F	Olecranon	Duration of the disease not mentioned. It presented 7 months after infliximab therapy was started	RA	Infliximab Methotrexate Prednisolone	Undetermined	Bursectomy	Complete resolution of bursitis. Later developed wrist arthritis

7	*C. parapsilosis* Shoulder synovial fluid Tissue culture	Jeong et al./2013 [10]	74/M	Subacromial, subdeltoid, and subcoracoid	Chronic, >18 months	None	None	Undetermined	Fluconazole (neither dose nor length of therapy specified) Surgical exploration with drainage, debridement, and bursectomy	Complete resolution

8	*C. parapsilosis* Bursal fluid	Current case	68/F	Olecranon	Acute, 1-2 weeks after bursa aspiration and corticosteroid injection	RA Infected ulcer	None	Corticosteroid injection	Caspofungin 50 mg IV daily for 2 weeks, followed by fluconazole 200 mg a day for 1 week	Persistence

9	*C. tropicalis* Bursal fluid Urine Blood	Murray et al./1976 [8]	77/M	Olecranon	Chronic	Bladder cancer Syphilis Sepsis	Neutropenia Urethral and venous catheters	Fungemia	Amphotericin B IV for 9 weeks Bursectomy	Complete resolution

10	*C. tropicalis* Blood Fluid from ruptured bursae	Wall et al./1982 [9]	48/M	First, knee septic arthritis; later, popliteal bursitis	Acute, 2 weeks after chemotherapy	Lymphocytic lymphoma	Chemotherapy Methotrexate Corticosteroids Neutropenia	Fungemia	Amphotericin B IV for 5 months Bursectomy and calf dissection	Complete resolution

11	Candida species No cultures, diagnosis done by histopathology	Khazzam et al./2005 [4]	65/M	Subacromial	Chronic, >4 months	Myocardial infarction	None	Corticosteroid injection	Voriconazole 200 mg twice daily for 6 weeks Bursectomy	Complete resolution

C: *Candida*; M: male; F: female; SLE: systemic lupus erythematosus; COPD: chronic obstructive pulmonary disease; IV: intravenous; RA: rheumatoid arthritis.
